# Degree-based topological indices and their graph energies in the QSPR analysis and ranking of UV-filter compounds

**DOI:** 10.3389/fchem.2026.1908317

**Published:** 2026-08-14

**Authors:** Merin Manuel, Parthiban Angamuthu

**Affiliations:** Department of Mathematics, School of Advanced Sciences, Vellore Institute of Technology, Vellore, Tamilnadu, India

**Keywords:** graph energy, MCDM, QSPR, SAW, topological indices, TOPSIS, UV-filters, VIKOR

## Abstract

Ultraviolet (UV) filters are essential ingredients in sunscreens and personal care products, and understanding their physicochemical properties is important for evaluating their performance and applicability. In this study, selected degree-based topological indices and their corresponding graph-energy descriptors were investigated as molecular descriptors for a set of commercially relevant UV-filter compounds. Quantitative structure-property relationship (QSPR) models were developed for molecular weight, complexity, XlogP, water solubility, topological polar surface area, refractivity, and polarizability. The results indicate that both classes of descriptors exhibit strong predictive potential for molecular weight, complexity, refractivity, and polarizability, while weaker relationships were observed for XlogP, water solubility, and polar surface area. The developed models were evaluated using regression analysis, leave-one-out cross-validation, and Monte Carlo validation, which produced consistent results for the well-performing properties. Furthermore, TOPSIS, SAW, and VIKOR methods were employed to rank the investigated UV-filters based on their physicochemical characteristics. The resulting rankings showed strong agreement, identifying Diethylhexyl Butamido Triazone, Ethylhexyl Triazone, and Bisoctrizole as the most promising candidates. The findings highlight the potential of degree-based topological descriptors and their graph energies for QSPR modeling, while the MCDM framework provides a systematic approach for the comparative evaluation and prioritization of UV-filter compounds.

## 1 Introduction

Solar energy is essential for sustaining life on Earth. Sunlight comprises electromagnetic radiation of different wavelengths, including infrared (IR), visible light, and ultraviolet (UV) radiation. Exposure to sunlight provides several physiological benefits, such as the synthesis of vitamin D in human skin. However, excessive exposure to UV radiation can have detrimental effects on human health. Solar ultraviolet radiation is commonly classified into three regions: UVA (320–400 nm), UVB (280–320 nm), and UVC (100–280 nm). UVA penetrates deeply into the skin and is primarily associated with photoaging, whereas UVB is responsible for erythema, sunburn, and direct DNA damage. UVC possesses the highest energy among the three regions but is largely absorbed by the Earth’s atmosphere and therefore does not normally reach the surface. Prolonged exposure to UV radiation has been linked to premature skin aging, hyperpigmentation, oxidative stress, DNA damage, and an increased risk of skin cancer (Giokas et al., 2007).

The need for protection against harmful solar radiation predates the development of modern sunscreens. Ancient civilizations employed natural substances and plant-derived extracts to reduce the adverse effects of sun exposure. The development of modern UV-filter technology began in the early twentieth century. In 1928, benzyl salicylate was identified as a compound capable of absorbing UVB radiation and was subsequently incorporated into the first commercial sunscreen, ‘Ambre Solaire’, introduced in 1935 (Jesus et al., 2022). Several decades later, avobenzone and related compounds emerged as effective UVA filters, significantly broadening the spectrum of photoprotection available in sunscreen formulations (Roelandis et al., 1983). Since then, UV-filters have become indispensable ingredients in sunscreens and personal care products, where they function by absorbing, reflecting, or scattering harmful ultraviolet radiation. Beyond cosmetic applications, UV-filters are also used in plastics, coatings, textiles, and packaging materials to enhance resistance to UV-induced degradation.

Despite their widespread use, increasing attention has been directed toward the potential human health and environmental impacts of UV-filters. Numerous studies have reported the presence of UV-filter compounds in human biological samples (Valle-Sistac et al., 2016) as well as in aquatic and marine organisms (Gago-Ferrero et al., 2012), raising concerns regarding their persistence, bioaccumulation, and possible toxicological effects. Consequently, several UV-filters have become subject to regulatory scrutiny and restrictions in different regions of the world. These concerns, combined with their extensive industrial and commercial applications, make UV-filters an important class of compounds for investigation from chemical, environmental, and pharmaceutical perspectives (Shetty et al., 2023; Ramos et al., 2015).

The physicochemical properties of UV-filters, including lipophilicity, water solubility, polarizability, and refractivity, play critical roles in determining their formulation characteristics, environmental fate, and biological interactions. Experimental determination of these properties is often time-consuming, expensive, and resource-intensive. Consequently, the development of reliable quantitative structure-property relationship (QSPR) models has attracted considerable interest as an efficient alternative for predicting molecular properties directly from structural information.

Chemical graph theory (Trinajstic, 2018) provides a powerful mathematical framework for investigating structure-property relationships in chemical compounds. Within this framework, a molecule is represented by a hydrogen-suppressed graph G=(V,E) , where the vertex set V corresponds to atoms and the edge set E represents chemical bonds. Structural information encoded in such molecular graphs can be quantified through numerical descriptors known as topological indices. These graph invariants have become indispensable tools in QSPR and related studies due to their ability to capture molecular features directly from chemical structure. The field traces its origins to the pioneering work of Harold Wiener in 1947, who introduced the path number, now known as the Wiener index (Wiener, 1947). Since then, numerous topological indices have been proposed and successfully employed for modeling a wide variety of physicochemical, biological, and pharmacological properties (Katritzky et al., 2010; Devillers and Balaban, 1999; Manuel and Angamuthu, 2025).

For a graph G , let $d_{\alpha }$ denote the degree of a vertex α∈V , that is, the number of vertices adjacent to α . Two vertices α and β are said to be adjacent, denoted by α∼β , whenever they are connected by an edge αβ∈E (West et al., 2001). Among the many classes of topological descriptors developed in the literature, degree-based topological indices (DTIs) are particularly attractive because of their simple formulation and proven effectiveness in structure-property modeling. In general, a degree-based topological index (Gutman, 2013) is constructed from the degrees of adjacent vertices and can be expressed in the following form.

$$TI\left(G\right)=\underset{\alpha \beta \in E\left(G\right)}{\sum }\zeta \left(d_{\alpha },d_{\beta }\right),$$

where ζ is a symmetric function defined on the degrees of the adjacent vertices α and β . Such indices have been extensively employed in QSPR investigations to model a wide range of physicochemical, thermodynamic, pharmacological, and biological properties of chemical compounds. Their success in capturing structural information has motivated the development of numerous variants and extensions, including spectral descriptors derived from graph matrices associated with topological indices.

A closely related branch of mathematical chemistry is spectral graph theory (Gutman et al., 2003), which studies graph structure through the eigenvalues of matrices associated with a graph. The fundamental matrix in this context is the adjacency matrix, whose entries indicate whether two vertices are adjacent. Building upon this concept, several weighted graph matrices have been introduced by replacing the adjacency entries with suitable functions of vertex degrees. Let $M=\left[m_{ij}\right]$ be a matrix associated with a graph invariant, where

$$m_{ij}=\left\{\begin{array}{cc} \zeta \left(d_{i},d_{j}\right), & \text{if }i∼j,\\ 0, & \text{otherwise}. \end{array}\right.$$

Here, $\zeta \left(d_{i},d_{j}\right)$ is a prescribed degree-dependent function that assigns weights to adjacent vertices. Thus, M may be viewed as a weighted generalization of the adjacency matrix corresponding to a particular topological descriptor.

The characteristic polynomial of M is defined by $\phi_{M}\left(\lambda \right)=\det\left(\lambda I-M\right)$ . Its roots $\lambda_{1},\lambda_{2},\ldots ,\lambda_{n}$ , called the eigenvalues of M , constitute the M -spectrum of the graph. Since M is symmetric, all eigenvalues are real. These spectral quantities encode important structural characteristics of the graph and provide an alternative representation of molecular topology. In analogy with the classical graph energy introduced by Gutman and Trinajstić, 1972), the energy associated with M is defined as

$$\xi \left(G\right)=\overset{n}{\underset{i=1}{\sum }}|\lambda_{i}|.$$

This quantity extends the notion of graph energy beyond the adjacency matrix and enables the construction of spectral descriptors, termed as graph-energy descriptors (GEDs), associated with different DTIs (Das et al., 2018). Consequently, distinct choices of the weighting function $\zeta \left(d_{i},d_{j}\right)$ generate different spectra and graph energies, offering additional molecular descriptors for structure-property investigations (Mondal et al., 2026; Raza and Munir, 2024).

In recent years, there has been a growing interest in the application of graph-theoretical descriptors for QSPR modeling across a wide range of chemical and pharmaceutical domains. Numerous studies have demonstrated the effectiveness of topological indices in investigating the structure-property relationships of diverse classes of compounds, including drugs for skin cancer (Pavithra and Ravi Sankar, 2026), depression (Godlin and Radha, 2026), asthma (Balasubramaniyan and Chidambaram, 2023), COVID-19 (Ugasini Preetha et al., 2024), and cardiovascular diseases (Arockiaraj et al., 2024), as well as fluoroquinolone antibiotics (Hanif, 2025) and food preservatives (Gayathri and Roy, 2025). But, most studies have concentrated on conventional graph descriptors, with comparatively fewer exploring GEDs derived from graph spectra. While both classes of descriptors originate from the same molecular graph, they describe different aspects of molecular structure. DTIs primarily reflect the connectivity of atoms within a molecule, whereas GEDs capture information related to the spectral characteristics of the corresponding graph matrices. As a result, GEDs have the potential to provide additional structural insight that complements traditional topological indices. Nevertheless, studies directly comparing the predictive performance of these two descriptor families within a common QSPR framework are still relatively scarce. In the present study, we consider the well-known degree-based indices $M_{1}$ , $M_{2}$ , R , H , ABC , GA , SO , and F , together with their corresponding graph energies listed in Table 1.

**TABLE 1:** Description of ζ related to DTIs and GEDs.

TI	ζ(u,v)	ξ
Atom-bond connectivity index (ABC) Estrada et al. (1998)	$\sqrt{\frac{u+v-2}{u\cdot v}}$	Atom-bond connectivity energy (ABCE) Chen (2018)
Randić index (R) Randic (1975)	$\frac{1}{\sqrt{u\cdot v}}$	Randić energy (RE) Gutman et al. (2014)
First Zagreb index $\left(M_{1}\right)$ Gutman and Trinajstić (1972)	u+v	First Zagreb energy $\left(M_{1}E\right)$ Rad et al. (2018)
Second Zagreb index $\left(M_{2}\right)$ Gutman and Trinajstić (1972)	u⋅v	Second Zagreb energy $\left(M_{2}E\right)$ Rad et al. (2018)
Harmonic index (H) Fajtlowicz (1988)	$\frac{2}{u+v}$	Harmonic energy (HE) Jahanbani and Raz (2019)
Forgotten index (F) Furtula et al. (2013)	$u^{2}+v^{2}$	Forgotten energy (FE) Mondal et al. (2026)
Geometric-arithmetic index (GA) Vukičević and Furtula (2009)	$\frac{2\sqrt{u\cdot v}}{u+v}$	Geometric-arithmetic energy (GAE) Rodríguez and Sigarreta (2016)
Sombor index (SO) Gutman (2021)	$\sqrt{u^{2}+v^{2}}$	Sombor energy (SOE) Ivan (2021)

Commercially important UV filters exhibit considerable structural diversity, ranging from simple aromatic systems to highly substituted and conjugated molecules. This diversity gives rise to a wide variation in physicochemical properties that influence their formulation, stability, and overall performance in sunscreen products. Developing reliable predictive models for these properties can therefore support the efficient screening and evaluation of UV-filter compounds while reducing the need for extensive experimental measurements. In view of this, this study investigates the applicability of DTIs and their corresponding GEDs for predicting the physicochemical properties of commercially important UV filters. In addition, the predicted properties are incorporated into a multi-criteria decision-making framework to facilitate the comparative ranking of these compounds based on selected physicochemical attributes.

## 2 Mathematical and computational methods

**FIGURE 1:**
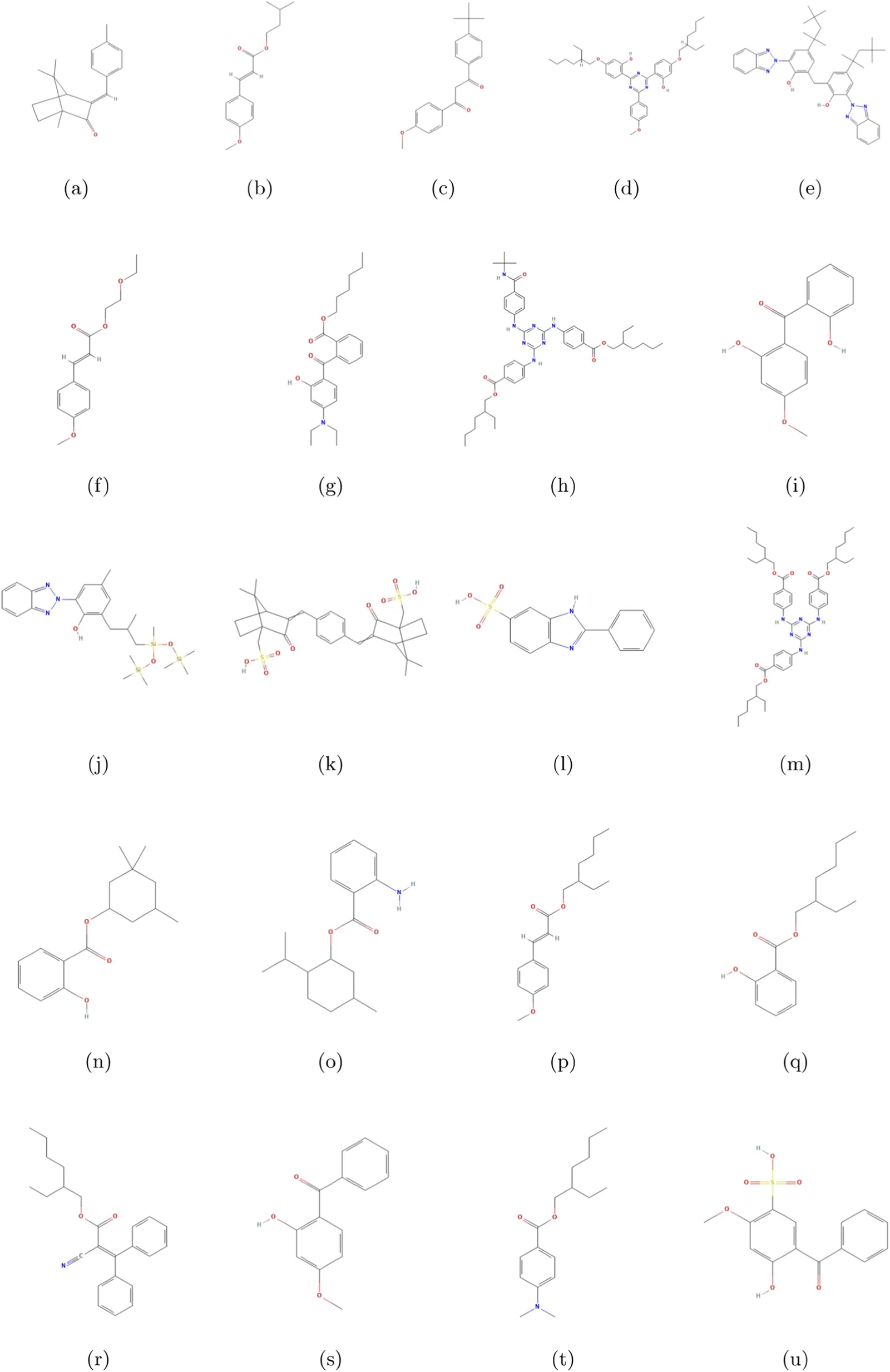
Chemical structures of the 21 compounds considered in the study: **(a)** 4-Methylbenzylidene Camphor **(b)** Amiloxate **(c)** Avobenzone **(d)** Bemotrizinol **(e)** Bisoctrizole **(f)** Cinoxate **(g)** Diethylamino Hydroxybenzoyl Hexyl Benzoate **(h)** Diethylhexyl Butamido Triazone **(i)** Dioxybenzone **(j)** Drometrizole Trisiloxane **(k)** Ecamsule **(l)** Ensulizole **(m)** Ethylhexyl Triazone **(n)** Homosalate **(o)** Meradimate **(p)** Octinoxate **(q)** Octisalate **(r)** Octocrylene **(s)** Oxybenzone **(t)** Padimate O **(u)** Sulisobenzone.

The present study considers a set of UV-filter compounds, namely, Avobenzone, Octocrylene, Octinoxate, Octisalate, Oxybenzone, Dioxybenzone, Sulisobenzone, Ensulizole, Homosalate, Padimate O, Meradimate, Cinoxate, Ecamsule, Drometrizole Trisiloxane, Diethylhexyl Butamido Triazone, Ethylhexyl Triazone, Diethylamino Hydroxybenzoyl Hexyl Benzoate, Bisoctrizole, Bemotrizinol, Amiloxate, and 4-Methylbenzylidene Camphor. Information regarding the selected UV-filters was obtained from the European Union Cosmetic Ingredients Database (CosIng) (European Commission, 2026). Molecular structures (Figure 1) and physicochemical property data (Table 2) were collected from the PubChem (NIH, 2026) and DrugBank (Knox et al., 2024) databases.

**TABLE 2:** Physicochemical properties of the UV-filters.

Name	Molecular weight	XLogP	Complexity	logS	Polar surface area	Refractivity	Polarizability
	MW (g/mol)		CX		PSA $\left({\text{Å}}^{2}\right)$	RT $\left({\text{m}}^{3}/\text{mol}\right)$	PT $\left({\text{Å}}^{3}\right)$
Avobenzone	310.4	4.8	405	−3.2	66.76	67.06	24.81
Octocrylene	361.5	7.1	510	−5.4	50.09	118.58	41.43
Octinoxate	290.4	5.3	304	−5.8	35.53	86.44	34.84
Octisalate	250.33	5.7	240	−4	46.53	72.21	28.8
Oxybenzone	228.24	3.6	258	−3.2	46.53	65.08	23.96
Dioxybenzone	244.24	3.3	292	−3.2	66.76	67.06	24.81
Sulisobenzone	308.31	2.2	462	−3.2	100.9	75.7	29.14
Ensulizole	274.3	2	414	−2.9	83.05	80.69	27.73
Homosalate	262.34	5	324	−3.8	46.53	74.65	29.86
Padimate O	277.4	5	270	−3.9	29.54	84.66	33.73
Meradimate	275.4	5.1	329	−4.7	52.32	81.97	31.56
Cinoxate	250.29	2.4	255	−3.7	44.76	70.08	27.39
Ecamsule	562.7	3.1	1230	−5	142.88	143.56	59.64
Drometrizole trisiloxane	501.8	12.4	613	−5.8	69.4	137.97	54.24
Diethylhexyl butamido triazone	766	11.8	1080	−5.9	156.46	223.95	87.63
Ethylhexyl triazone	823.1	14.5	1030	−6.7	153.66	240.34	97.67
Diethylamino hydroxybenzoyl hexyl benzoate	397.5	6.5	505	−4.8	66.84	117.64	45.58
Bisoctrizole	658.9	12.8	989	−6.2	101.88	220.17	75.8
Bemotrizinol	627.8	10.4	761	−6.3	106.82	216.4	75.71
Amiloxate	248.32	3.9	263	−4.5	35.53	72.71	28.33
4-Methylbenzylidene camphor	254.4	4.5	423	−4.7	17.07	79.68	30.58

The physicochemical properties investigated in this study include molecular weight (MW, g/mol ), octanol/water partition coefficient (XlogP), water solubility (LogS), topological polar surface area (PSA, ${\text{A}{}^{\circ}}^{2}$ ), molar refractivity (RT, ${\text{m}}^{3}/\text{mol}$ ), complexity (CX), and molecular polarizability $\left(PT,{\text{A}{}^{\circ}}^{3}\right)$ . These properties were selected because of their relevance to the formulation performance, environmental behavior, and biological interactions of UV-filter compounds.

All computations were performed in the Jupyter Notebook environment using Python (version 7.2.2). Molecular structures represented by SMILES strings were converted into molecular graphs, from which the DTIs and their corresponding GEDs (Table 3) were calculated. The computational workflow utilized the libraries NumPy, Pandas, NetworkX, RDKit, Statsmodels, Scikit-learn, and Matplotlib for data processing, graph-theoretical computations, statistical analysis, model development, validation, and visualization.

**TABLE 3:** Calculated topological descriptors of the UV-filters.

Calculated DTIs							
Name	$M_{1}$	$M_{2}$	R	H	ABC	GA	SO	F
Avobenzone	116	132	10.81	10.22	17.52	22.84	85.39	312
Octocrylene	126	143	13.21	12.93	19.75	27.51	90.40	302
Octinoxate	92	99	10.19	9.90	14.96	20.47	66.50	216
Octisalate	80	88	8.69	8.40	12.87	17.50	57.89	192
Oxybenzone	84	97	8.22	7.97	12.83	17.53	60.67	210
Dioxybenzone	90	105	8.63	8.30	13.60	18.40	65.25	230
Sulisobenzone	108	126	9.84	9.29	16.03	20.92	79.45	296
Ensulizole	104	122	9.04	8.65	15.18	20.19	76.05	282
Homosalate	98	111	8.87	8.33	14.71	18.92	72.49	268
Padimate O	90	99	9.58	9.20	14.39	19.32	65.43	220
Meradimate	100	115	9.49	9.03	15.23	20.17	72.94	258
Cinoxate	78	83	8.76	8.53	12.84	17.59	56.28	180
Ecamsule	228	286	17.35	15.98	30.91	39.15	170.04	708
Drometrizole trisiloxane	178	203	15.14	14	26.03	32.65	133.04	514
Diethylhexyl butamido triazone	276	310	26.89	25.90	42.40	57.05	200.92	700
Ethylhexyl triazone	288	324	29.17	28.40	44.75	61.53	207.86	702
Diethylamino hydroxybenzoyl hexyl benzoate	138	159	14.03	13.60	21.30	29.24	99.56	342
Bisoctrizole	278	330	22.79	21.37	39.54	51.04	205.68	798
Bemotrizinol	228	263	22.38	21.80	34.66	47.90	164.35	566
Amiloxate	80	85	8.61	8.27	13.06	17.36	58.31	192
4-Methylbenzylidene camphor	110	138	8.86	8.32	15.25	19.91	81.03	324

Calculated GEDs							
Name	$M_{1}E$	$M_{2}E$	RE	HE	ABCE	GAE	SOE	FE
Avobenzone	133.53	150.64	13.35	12.58	20.87	27.03	98.31	351.74
Octocrylene	152.24	168.73	17.18	16.75	24.69	34.19	109.38	356.93
Octinoxate	112.33	118.61	13.45	13.01	18.86	25.70	81.28	258.82
Octisalate	96.08	104.03	11.28	10.83	16	21.58	69.61	226.31
Oxybenzone	99.78	113.16	10.53	10.13	15.80	21.37	72.20	244.87
Dioxybenzone	104.88	120.70	10.89	10.38	16.45	21.98	76.18	263.61
Sulisobenzone	122.78	141.70	12.12	11.38	18.94	24.52	90.35	328.83
Ensulizole	118.28	138.18	10.69	10.26	17.64	23.46	86.35	314.66
Homosalate	108.64	124.10	10.29	9.60	16.64	21.30	80.25	293.12
Padimate O	107.82	116.47	12.40	11.84	17.87	23.76	78.57	259.13
Meradimate	114.48	131.22	11.44	10.81	17.91	23.42	83.60	292.47
Cinoxate	96.29	100.67	11.56	11.22	16.31	22.25	69.55	218.26
Ecamsule	241.60	300.79	19.86	18.18	34.21	42.64	180.46	735.79
Drometrizole trisiloxane	192.52	220.71	17.10	15.75	28.74	35.91	143.69	548.06
Diethylhexyl butamido triazone	320.07	356.11	33.14	31.84	50.44	67.69	232.96	798.58
Ethylhexyl triazone	340.61	377.43	36.95	35.80	54.49	74.50	246.11	817.72
Diethylamino hydroxybenzoyl hexyl benzoate	162.99	184.17	17.98	17.30	26.14	35.48	117.84	396.44
Bisoctrizole	303.52	360.94	26.03	24.34	44.17	56.78	224.28	857.97
Bemotrizinol	268.28	304.85	28.28	27.43	42.02	57.79	193.55	655.16
Amiloxate	96.01	101.09	10.92	10.45	16.04	21.25	70.01	227.29
4-Methylbenzylidene camphor	121.04	149.99	10.77	10	17.76	22.62	89.43	348.08

Linear regression models were developed using the calculated descriptors as independent variables and the selected physicochemical properties as dependent variables. Statistical significance and goodness-of-fit measures were obtained using the Statsmodels package.

Model validation was performed through internal and external validation procedures, including Monte Carlo cross-validation based on repeated random training-test splits. Predictive performance was assessed using standard statistical metrics.

The ranking of UV-filter compounds was carried out using MCDM techniques. The consistency of the resulting rankings was further examined using Spearman’s rank correlation coefficient, Kendall’s tau coefficient, and average rank difference (ARD). Graphical representations and all reported figures were generated using Python-based visualization tools.

**FIGURE 2:**
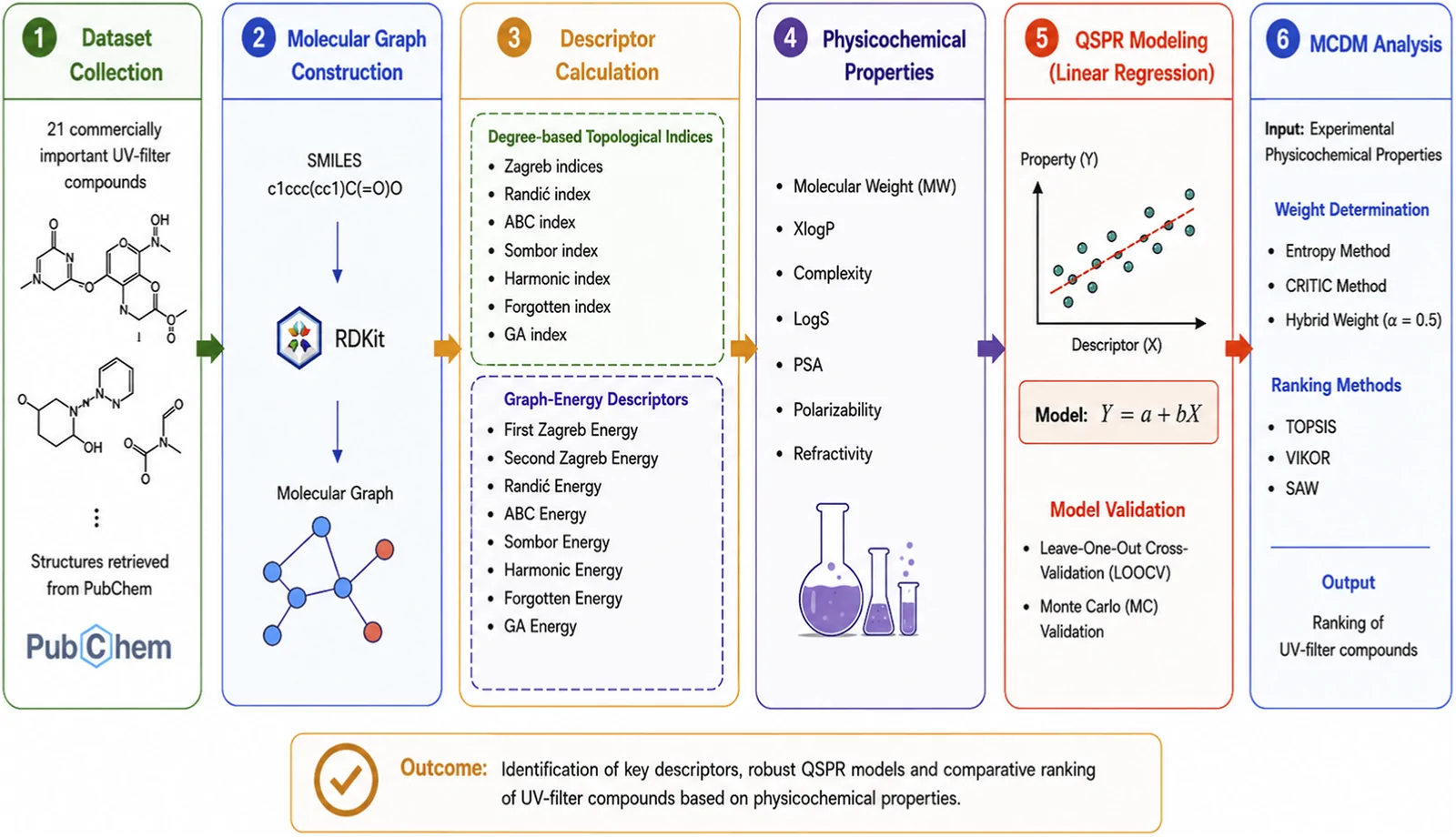
Overall workflow adopted in this study.

Figure 2 presents the overall workflow of the proposed methodology, highlighting the sequential steps involved in descriptor calculation, QSPR analysis, model validation, and MCDM-based ranking.

## 3 Analysis of the structure-property relations

### 3.1 Correlation analysis

**FIGURE 3:**
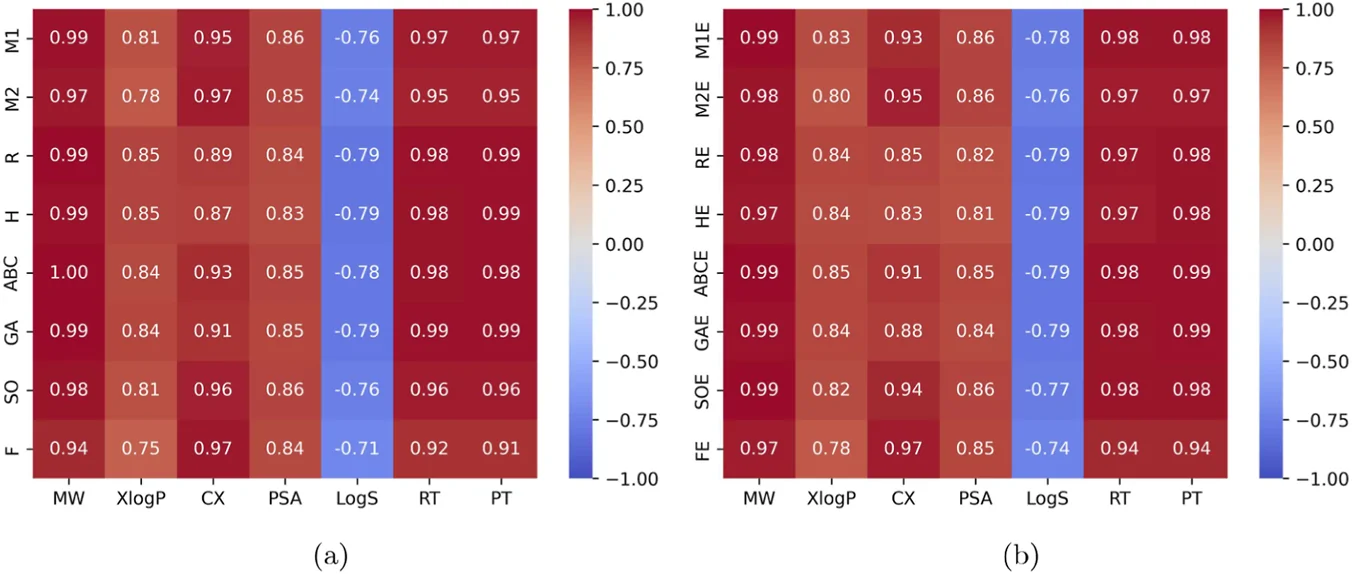
Correlation heatmap of the properties of UV-filters with **(a)** DTIs **(b)** GEDs.

A fundamental step in QSPR studies is the assessment of the relationship between molecular descriptors and experimentally determined physicochemical properties. Correlation analysis provides a preliminary indication of the ability of a descriptor to capture structural features relevant to a particular property. In general, stronger correlations suggest greater potential for the descriptor to serve as an effective predictor in subsequent regression modeling. The Pearson correlation coefficients between the investigated DTIs, their corresponding graph energies, and the selected physicochemical properties are presented in the heatmap shown in Figure 3.

Inspection of Figure 3 reveals noticeable differences in the predictive potential of the descriptors across the considered properties. Among the seven properties examined, XlogP, PSA, and LogS exhibit comparatively weaker correlations with both the DTIs and the associated graph energies. In particular, the correlation coefficients for these properties remain below 0.86, while LogS displays predominantly negative correlations, indicating an inverse relationship with increasing descriptor values. These observations suggest that the structural information encoded by the investigated descriptors is less directly associated with lipophilicity, polar surface area, and aqueous solubility than with the remaining properties.

In contrast, the descriptors show very strong correlations with molecular weight, complexity, refractivity, and polarizability, with many correlation coefficients exceeding 0.90 and several approaching 0.99. The particularly high correlations observed for molecular weight are expected, as DTIs are strongly influenced by molecular size and connectivity, both of which generally increase with molecular mass. Similar trends are observed for the corresponding GEDs, indicating that the spectral quantities preserve much of the structural information contained in the original indices.

Among the descriptors considered, the F -index and its associated graph energy generally exhibit comparatively weaker correlations with most properties. An exception is observed for molecular complexity, for which these descriptors demonstrate some of the strongest correlations. Furthermore, substantial correlations among many of the descriptors themselves indicate the presence of shared structural information, reflecting the fact that several degree-based indices are derived from related graph-theoretical concepts.

To reduce redundancy and avoid the inclusion of highly correlated descriptors in subsequent modeling, only the best-performing descriptor pair (topological index and corresponding graph energy) for each physicochemical property was selected. The selected descriptors are listed in Table 4. Overall, the results indicate that the graph energies exhibit correlation patterns remarkably similar to those of their parent topological indices, although the original DTIs generally provide marginally stronger correlations with the investigated properties.

**TABLE 4:** Selected descriptor-property pairs for regression analysis.

Name	MW	XLogP	CX	logS	PSA	RT	PT
DTI	ABC	R	F	R	$M_{1}$	GA	R
GED	ABCE	ABCE	FE	RE	$M_{2}E$	ABCE	ABCE

### 3.2 Regression analysis

To assess the predictive capability of the selected descriptors, simple linear regression models (Montgomery et al., 2021) of the form P=wD+k were developed, where P denotes the physicochemical property under investigation, D represents the topological descriptor, w is the regression coefficient (slope), and k is the intercept. The quality of the resulting models was evaluated using several statistical measures, including the coefficient of determination $\left(R^{2}\right)$ , adjusted $R^{2}$ , root mean square error (RMSE), mean absolute error (MAE), the F-statistic, and the associated p-value. To further examine model stability and predictive performance, leave-one-out cross-validation (LOO-CV) (Stone, 1974) was performed, and the corresponding cross-validated coefficient of determination $\left(Q^{2}\right)$ and prediction error metrics were calculated. The regression statistics obtained for the DTIs and their corresponding graph energies are summarized in Table 5.

**TABLE 5:** Regression analysis of the properties of UV-filters.

DTIs							
Metrics	MW - ABC	XLogP - R	CX - F	logS - R	PSA - $M_{1}$	RT - GA	PT - R
$R^{2}$	0.991	0.729	0.942	0.510	0.734	0.973	0.982
Adj_ $R^{2}$	0.991	0.715	0.938	0.484	0.720	0.971	0.981
F-stat	2179.04	51.09	306.07	19.76	52.42	675.90	1058.58
p-value	4.54E-21	8.56E-07	3.57E-13	2.78E-04	7.14E-07	2.58E-16	3.98E-18
RMSE	17.03	1.93	73.13	0.81	20.45	9.64	2.93
MAE	12.83	1.30	51.16	0.67	15.85	7.14	2.09
Intercept	13.592	−0.374	−32.956	−3.047	4.495	−3.644	−2.377
Slope	17.615	0.496	1.491	−0.004	0.480	4.141	3.433
$Q^{2}$ -LOO	0.988	0.685	0.913	0.415	0.665	0.964	0.980
RMSECV	20.01	2.08	89.13	0.89	22.94	10.99	3.15

GEDs							
Metrics	MW - ABCE	XLogP - ABCE	CX - FE	logS - RE	PSA - $M_{2}E$	RT - ABCE	PT - ABCE
$R^{2}$	0.990	0.714	0.935	0.628	0.737	0.970	0.980
Adj_ $R^{2}$	0.989	0.699	0.931	0.609	0.723	0.968	0.979
F-stat	1811.42	47.55	272.06	32.12	53.17	604.61	923.06
p-value	2.59E-20	1.41E-06	1.02E-12	1.83E-05	6.46E-07	7.23E-16	1.43E-17
RMSE	18.66	1.98	77.28	0.71	20.34	10.18	3.14
MAE	12.01	1.33	48.57	0.58	15.69	7.54	2.30
Intercept	5.356	−0.348	−53.032	−2.666	4.151	−6.867	−2.600
Slope	15.153	0.261	1.373	−0.118	0.369	4.776	1.819
$Q^{2}$ -LOO	0.988	0.667	0.909	0.559	0.670	0.959	0.977
RMSECV	20.07	2.14	91.46	0.77	22.77	11.75	3.37

In general, a reliable QSPR model is characterized by high values of $R^{2}$ , adj_ $R^{2}$ , and $Q^{2}$ , indicating that a substantial proportion of the variation in the property is explained by the descriptor. A small difference between $R^{2}$ and $Q^{2}$ indicates model robustness and suggests that the predictive performance is maintained upon validation. Conversely, lower values of RMSE and MAE indicate improved predictive accuracy. The statistical significance of each regression model is assessed through the F-statistic and its associated p-value, with p<0.05 generally considered indicative of a statistically significant linear relationship between the descriptor and the property.

The regression statistics reported in Table 5 largely support the trends observed in the correlation analysis. Among the investigated properties, XlogP, LogS, and PSA are comparatively less well described by the selected descriptors, as evidenced by their lower coefficients of determination, generally below 0.75. Nevertheless, the corresponding models remain statistically significant, indicating the existence of meaningful, albeit weaker, linear relationships between these properties and the molecular descriptors. Interestingly, the GEDs exhibit slightly improved predictive performance for LogS and PSA when compared with their parent topological indices.

In contrast, molecular weight, complexity, refractivity, and polarisability demonstrate strong linear relationships with the investigated descriptors. For these properties, the regression models yield high coefficients of determination accompanied by large F-statistics and low prediction errors. The conventional DTIs consistently provide marginally better performance than the corresponding GEDs, although the differences are generally small. The highest predictive accuracy is observed for molecular weight, for which $R^{2}$ values approaching 0.99. Similarly, refractivity, polarizability, and complexity exhibit excellent fits, with $R^{2}$ values reaching approximately 0.97, 0.98, and 0.94, respectively.

The error measures further support the reliability of these models, remaining relatively small compared with the observed ranges of the corresponding physicochemical properties. Moreover, the cross-validated $Q^{2}$ values are in close agreement with the original $R^{2}$ values, suggesting that the developed models possess satisfactory robustness and are not substantially affected by overfitting.

**FIGURE 4:**
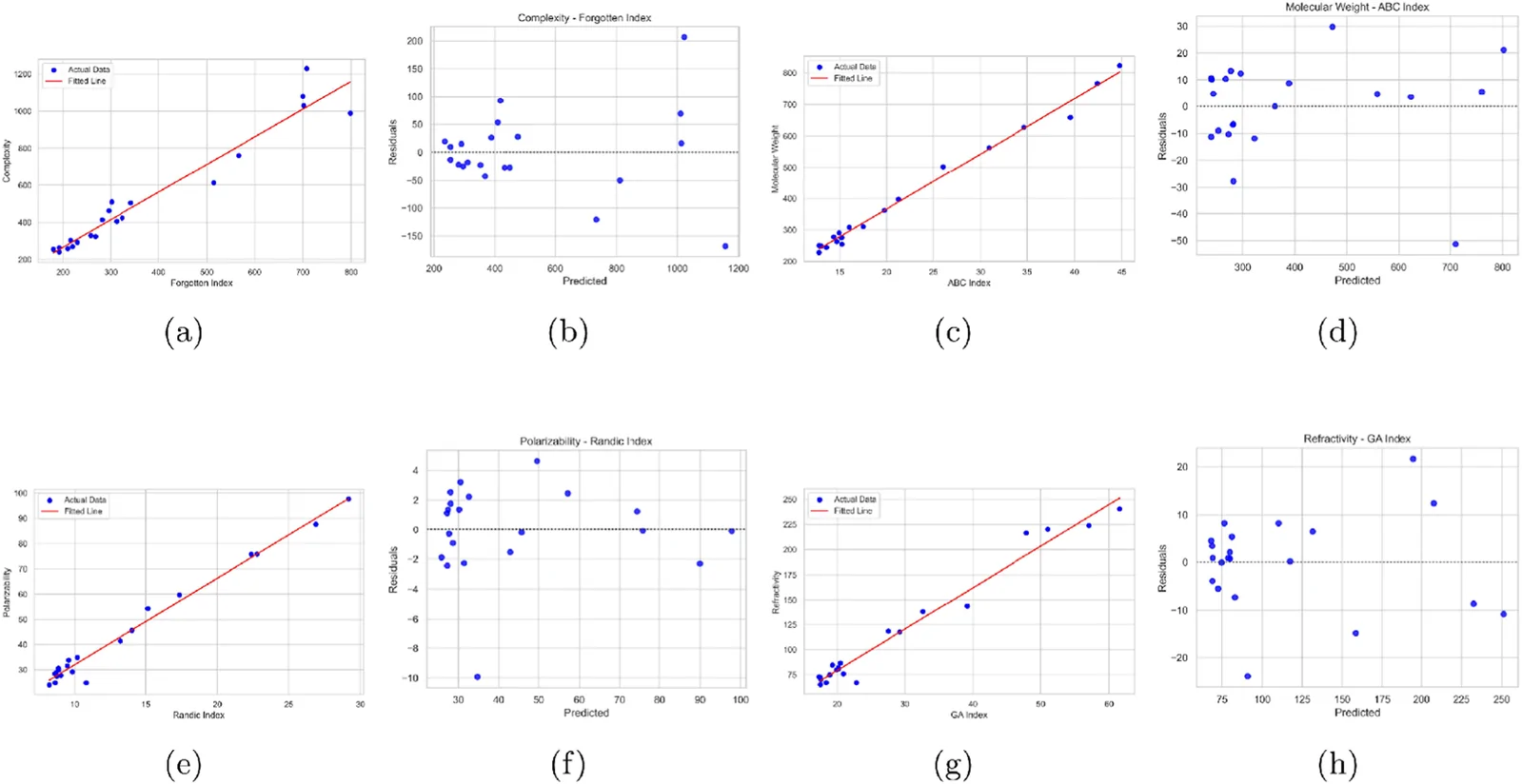
Scatter and residual plots corresponding to DTIs: **(a)** Scatter plot: complexity and F -index **(b)** Residual plot: complexity and F -index **(c)** Scatter plot: molecular weight and ABC -index **(d)** Residual plot: molecular weight and ABC -index **(e)** Scatter plot: polarizability and R -index **(f)** Residual plot: polarizability and R -index **(g)** Scatter plot: refractivity and GA -index **(h)** Residual plot: refractivity and GA -index.

**FIGURE 5:**
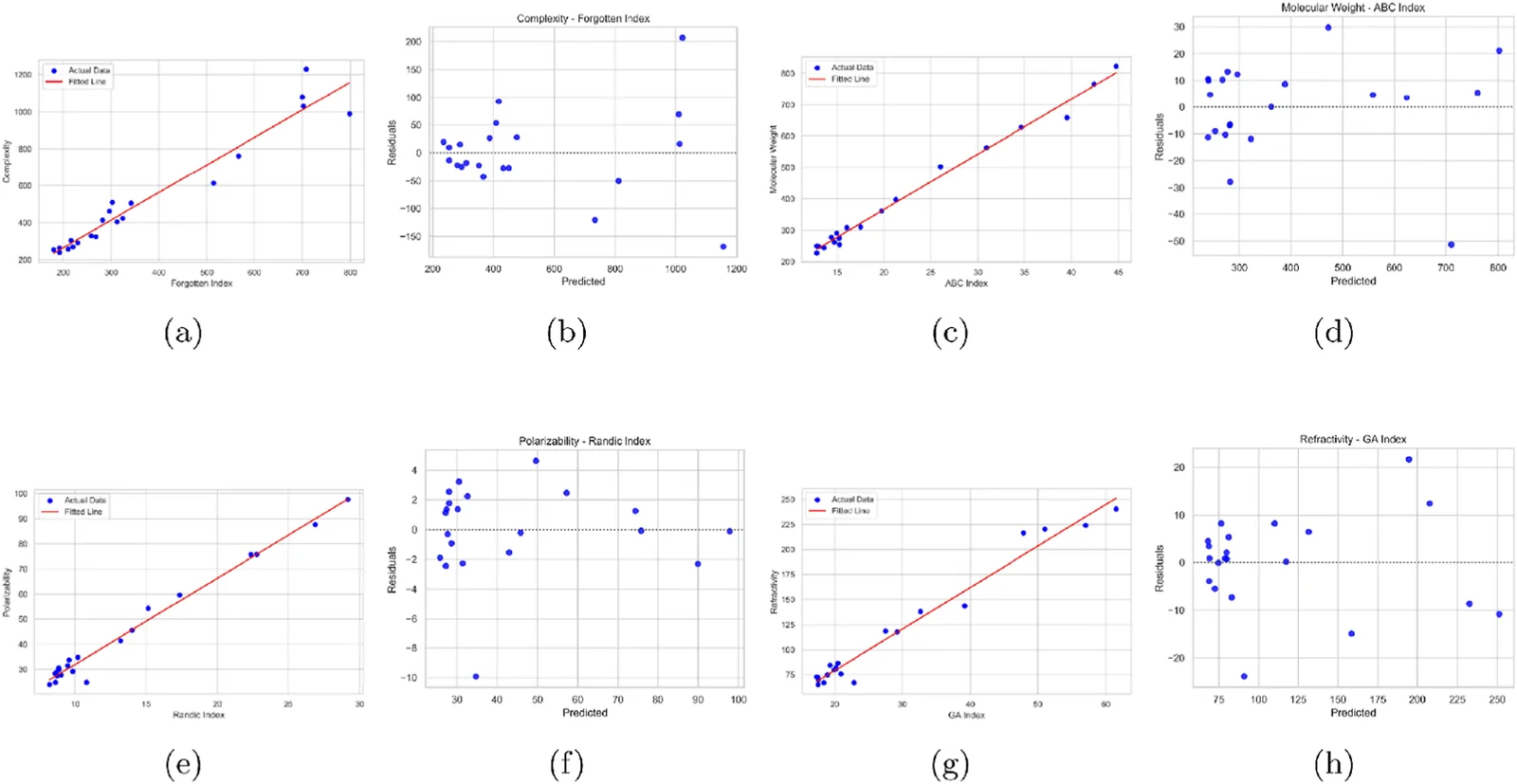
Scatter and residual plots corresponding to GEDs: **(a)** Scatter plot: complexity and FE **(b)** Residual plot: complexity and FE **(c)** Scatter plot: molecular weight and ABCE **(d)** Residual plot: molecular weight and ABCE **(e)** Scatter plot: polarizability and ABCE **(f)** Residual plot: polarizability and ABCE **(g)** Scatter plot: refractivity and ABCE **(h)** Residual plot: refractivity and ABCE

The regression performance of the best-performing models is further illustrated through the scatter and residual plots presented in Figures Figures 4, 5. The scatter plots reveal a strong linear association between the predicted and experimental values, with most observations lying close to the fitted regression line. The residual plots show that the residuals are distributed approximately randomly around zero without any obvious systematic pattern, indicating that the linear models adequately capture the underlying relationships. Although a small number of observations deviate noticeably from the general trend, such isolated points are expected in datasets of chemically diverse UV-filter compounds and do not significantly affect the overall quality of the models.

### 3.3 Monte carlo validation

In addition to leave-one-out cross-validation, Monte Carlo validation (Xu et al., 2004) was performed to further assess the robustness and predictive reliability of the developed QSPR models. Unlike LOO-CV, which systematically omits a single observation at a time, Monte Carlo validation repeatedly partitions the dataset into independent training and test subsets, thereby providing a more realistic evaluation of model performance under varying data compositions. This procedure enables the assessment of model stability, predictive consistency, and generalizability while reducing the influence of any particular train-test partition.

For the present study, 500 independent iterations were carried out using an 80:20 training-to-test ratio. For each iteration, the predictive coefficient of determination $\left(R_{\text{pred}}^{2}\right)$ and the corresponding prediction error metrics were recorded. The resulting statistics for the models based on DTIs and GEDs are summarized in Table 6.

**TABLE 6:** Monte carlo validation of the regression analysis.

Monte Carlo validation			Iterations: 500			Train/Test split: 80/20		
DTIs								
Metrics	​	MW - ABC	XLogP - R	CX - F	logS - R	PSA - $M_{1}$	RT - GA	PT - R
$R_{\text{pred}}^{2}$	Mean	0.946	0.232	0.866	0.290	0.311	0.848	0.873
​	SD	0.360	1.697	0.116	0.727	0.711	0.854	0.706
​	$R_{\text{pred}}^{2}$ > 0.5	493 (98.6%)	313 (62.6%)	495 (99%)	240 (48%)	279 (55.8%)	485 (97%)	482 (96.4%)
​	$R_{\text{pred}}^{2}$ > 0.8	487 (97.4%)	191 (38.2%)	395 (79%)	41 (8.2%)	82 (16.4%)	460 (92%)	464 (92.8%)
RMSEP	Mean	19.40	1.95	83.57	0.75	22.90	10.84	2.89

GEDs								
Metrics	​	MW - ABCE	XLogP - ABCE	CX - FE	logS - RE	PSA - $M_{2}E$	RT - ABCE	PT - ABCE
$R_{\text{pred}}^{2}$	Mean	0.956	0.162	0.878	0.260	0.326	0.835	0.856
​	SD	0.194	1.917	0.101	0.775	0.717	0.966	0.808
​	$R_{\text{pred}}^{2}$ > 0.5	496 (99.2%)	302 (60.4%)	497 (99.4%)	233 (46.6%)	281 (56.2%)	483 (96.6%)	476 (95.2%)
​	$R_{\text{pred}}^{2}$ > 0.8	489 (97.8%)	187 (37.4%)	434 (86.8%)	36 (7.2%)	82 (16.4%)	457 (91.4%)	461 (92.2%)
RMSEP	Mean	19.36	1.99	82.85	0.77	22.73	11.59	3.07

The Monte Carlo results are largely consistent with the conclusions drawn from the regression analysis and LOO-CV. Among the investigated properties, molecular weight, complexity, refractivity, and polarizability exhibit the strongest predictive performance for both classes of descriptors. In particular, the DTI-based model for molecular weight achieved an average predictive coefficient of $R_{\text{pred}}^{2}=0.946$ , with 98.6% of the generated models yielding $R_{\text{pred}}^{2}>0.5$ and 97.4% producing $R_{\text{pred}}^{2}>0.8$ . Similarly, the corresponding energy-based model achieved an average predictive coefficient of $R_{\text{pred}}^{2}=0.956$ , with 99.2% and 97.8% of the generated models exceeding the thresholds of 0.5 and 0.8, respectively.

A similar pattern is observed for CX, RT, and PT, where a substantial majority of the generated models achieve $R_{\text{pred}}^{2}>0.8$ , demonstrating strong predictive stability across repeated random partitions of the dataset. For MW and CX, the GEDs provide a modest improvement over the conventional degree-based indices, indicating that the spectral information encoded by the energy measures may offer additional predictive value for these properties.

In contrast, XlogP, LogS, and PSA exhibit noticeably weaker predictive performance. This observation is consistent with the correlation and regression analyses, which likewise indicated weaker relationships between these properties and the investigated descriptors. The larger variability in predictive performance observed for these models suggests that the structural information captured by the selected descriptors is less effective in explaining properties associated with lipophilicity, polarity, and aqueous solubility.

Overall, the Monte Carlo validation results support the trends observed in the regression and LOO-CV analyses. The favorable predictive performance obtained for molecular weight, complexity, refractivity, and polarizability across a large proportion of the random partitions indicates that the corresponding models possess reasonable predictive stability. While occasional train-test splits resulted in lower predictive coefficients, such variations are expected given the size and diversity of the dataset. Taken together, the validation results suggest that the investigated descriptors capture structural information relevant to these properties and may be useful for QSPR modeling within the studied class of UV-filter compounds.

## 4 Ranking of UV-filters using MCDM techniques

To comparatively evaluate the suitability of the investigated UV-filter compounds and identify promising candidates for further experimental and formulation studies, a multi-criteria decision-making (MCDM) framework was employed. The analysis was based on selected physicochemical descriptors, namely, molecular weight, XlogP, molecular complexity, logS, topological polar surface area, molar refractivity, and polarizability. These descriptors collectively capture important molecular characteristics related to size, lipophilicity, structural complexity, solubility, polarity, and optical behavior.

Prior to analysis, the descriptor values were normalized to eliminate differences in measurement scales. Criterion weights were then determined using a hybrid Entropy-CRITIC weighting scheme. The Entropy method (Hwang and Yoon, 2012) evaluates the information content of each criterion based on data dispersion, whereas the CRITIC (Criteria Importance Through Intercriteria Correlation) method (Diakoulaki et al., 1995) accounts for both criterion variability and inter-criterion relationships. The combination of these objective weighting approaches reduces subjective bias while considering multiple characteristics of the dataset.

The normalized and weighted criteria were subsequently analyzed using the Simple Additive Weighting (SAW) (Hwang and Yoon, 2012), Technique for Order Preference by Similarity to Ideal Solution (TOPSIS) (Hwang and Yoon, 2012), and VlseKriterijumska Optimizacija I Kompromisno Resenje (VIKOR) (Opricovic and Tzeng, 2004) methods.

The SAW method is one of the simplest and most widely used MCDM techniques. It evaluates each alternative by calculating the weighted sum of normalized criterion values, where larger scores indicate more desirable alternatives. The overall score of the $i^{\text{th}}$ alternative is calculated as

$$S_{i}=\overset{n}{\underset{j=1}{\sum }}w_{j}r_{ij},$$

where $S_{i}$ is the overall score of alternative i , $w_{j}$ is the weight assigned to the $j^{\text{th}}$ criterion, $r_{ij}$ is the normalized value of the $j^{\text{th}}$ criterion for the $i^{\text{th}}$ alternative, and n denotes the total number of criteria. Alternatives with higher values of $S_{i}$ are preferred.

TOPSIS ranks alternatives according to their relative closeness to an ideal solution. After normalization and weighting of the decision matrix, the positive ideal solution (best criterion values) and negative ideal solution (worst criterion values) are identified. Each alternative is then ranked based on its relative distance from these ideal solutions, with higher closeness coefficients indicating better overall performance. The relative closeness coefficient is computed as

$$C_{i}=\frac{D_{i}^{-}}{D_{i}^{+}+D_{i}^{-}},$$

where $D_{i}^{+}$ and $D_{i}^{-}$ denote the Euclidean distances of the $i^{\text{th}}$ alternative from the positive ideal solution and negative ideal solution, respectively. A larger value of $C_{i}$ indicates that the alternative is closer to the ideal solution and hence ranks higher.

VIKOR identifies compromise solutions by simultaneously considering overall utility and individual criterion regret. The VIKOR index is determined by

$$Q_{i}=v\left(\frac{S_{i}-S^{*}}{S^{-}-S^{*}}\right)+\left(1-v\right)\left(\frac{R_{i}-R^{*}}{R^{-}-R^{*}}\right),$$

where $S_{i}$ represents the group utility measure, $R_{i}$ denotes the individual regret measure, $S^{*}$ and $R^{*}$ are the best values, $S^{-}$ and $R^{-}$ are the worst values, and v is the weight of the majority criterion. Alternatives with smaller $Q_{i}$ values are considered more desirable.

The entropy of the j-th criterion is given by

$$E_{j}=-k\overset{m}{\underset{i=1}{\sum }}p_{ij}\ln\left(p_{ij}\right),$$

where $p_{ij}$ is the normalized value of criterion j for alternative i , m is the number of alternatives, and k is the reciprocal of ln(m) . The corresponding entropy weight is

$$w_{j}^{\left(E\right)}=\frac{1-E_{j}}{\overset{n}{\underset{j=1}{\sum }}\left(1-E_{j}\right)}.$$

Criteria with greater information content (lower entropy) receive higher weights.

The CRITIC method is an objective weighting technique that determines criterion weights based on both the variation of individual criteria and the degree of conflict among them. Criteria exhibiting greater variability and lower correlation with other criteria receive larger weights. The information content of each criterion is calculated as

$$C_{j}=\sigma_{j}\overset{n}{\underset{k=1}{\sum }}\left(1-r_{jk}\right),$$

where $\sigma_{j}$ is the standard deviation of criterion j and $r_{jk}$ is the correlation coefficient between criteria j and k . The CRITIC weight is then obtained as

$$w_{j}^{\left(C\right)}=\frac{C_{j}}{\overset{n}{\underset{j=1}{\sum }}\left(C_{j}\right)}.$$

This method assigns larger weights to criteria exhibiting greater variability and lower correlation with other criteria.

To balance the strengths of two objective weighting approaches, the final criterion weights were obtained by combining the Entropy and CRITIC weights using an equal-weight strategy. This hybrid approach simultaneously considers the amount of information contained in each criterion and the interdependence among criteria, leading to a more balanced and robust weighting scheme for the subsequent MCDM analysis. The hybrid weight vector was computed as $W=\alpha w_{j}^{\left(E\right)}+\left(1-\alpha \right)w_{j}^{\left(C\right)}$ , where α=0.5 , assigning equal contributions to the entropy and CRITIC weighting methods.

It should be noted that the objective of the MCDM analysis was not to establish an absolute measure of UV-filter performance, but rather to provide a comparative assessment of the investigated compounds based on their overall physicochemical profiles. Consequently, the resulting rankings should be interpreted only with respect to the selected physicochemical criteria and not as a comprehensive evaluation of the overall suitability of the compounds. Important practical considerations, including photostability, toxicological profile, endocrine activity, formulation compatibility, environmental impact, and regulatory requirements, were beyond the scope of the present study and should be incorporated in future comprehensive assessments.

To evaluate the consistency and reliability of the obtained rankings, Spearman’s rank correlation coefficient (Hollander et al., 2013), Kendall’s tau coefficient (Hollander et al., 2013), and the Average Rank Difference (ARD) (Triantaphyllou, 2000) were calculated. These measures provide insight into the degree of agreement among the ranking methods and the robustness of the resulting compound prioritization.

The weighting procedure produced a relatively balanced distribution of weights among the selected physicochemical properties (Table 7). Molecular complexity received the highest weight (0.15966), followed by XlogP (0.14965) and refractivity (0.14914), indicating their comparatively greater contribution to differentiating the investigated UV-filters. However, the differences among the weights were small, suggesting that all selected properties provide meaningful and complementary information for the ranking process.

**TABLE 7:** Hybrid weights obtained using Entropy - CRITIC method.

MW	XLogP	CX	logS	PSA	RT	PT
0.13297	0.14965	0.15966	0.13342	0.14382	0.14914	0.13134

The rankings obtained using the TOPSIS, SAW, and VIKOR methods are presented in Table 8. Although minor differences in individual rank positions are observed, the overall ranking patterns remain largely similar across the three approaches. Among the investigated compounds, Diethylhexyl Butamido Triazone, Ethylhexyl Triazone, and Bisoctrizole consistently occupy the top positions, whereas Octinoxate, Cinoxate, and Amiloxate are generally placed near the bottom of the rankings. This overall agreement suggests that the relative performance of the compounds is not strongly influenced by the choice of ranking method.

**TABLE 8:** Comparison and ranking of UV-filters using various MCDM methods.

Name	TOPSIS	SAW	VIKOR	TOPSIS rank	SAW rank	VIKOR rank
Avobenzone	0.2583	0.2559	0.8116	11	11	10
Octocrylene	0.2901	0.2908	0.5154	8	8	8
Octinoxate	0.1537	0.1519	0.9032	21	20	16
Octisalate	0.2049	0.1891	0.9655	15	16	19
Oxybenzone	0.2118	0.1753	0.9504	14	17	18
Dioxybenzone	0.2294	0.2049	0.8816	12	13	15
Sulisobenzone	0.2889	0.2837	0.7897	9	9	9
Ensulizole	0.2702	0.2599	0.8277	10	10	11
Homosalate	0.2128	0.2080	0.8347	13	12	12
Padimate O	0.2048	0.1970	0.9180	16	14	17
Meradimate	0.1917	0.1965	0.8361	17	15	13
Cinoxate	0.1869	0.1564	0.9683	18	19	21
Ecamsule	0.5600	0.5674	0.4909	5	5	7
Drometrizole trisiloxane	0.4649	0.4474	0.2783	6	6	4
Diethylhexyl butamido triazone	0.7824	0.7935	0.0578	1	2	1
Ethylhexyl triazone	0.7765	0.8314	0.2732	2	1	3
Diethylamino hydroxybenzoyl hexyl benzoate	0.3215	0.3358	0.4898	7	7	6
Bisoctrizole	0.6947	0.6758	0.2342	3	3	2
Bemotrizinol	0.6235	0.6015	0.3184	4	4	5
Amiloxate	0.1551	0.1415	0.9679	20	21	20
4-Methylbenzylidene camphor	0.1723	0.1597	0.8498	19	18	14

**FIGURE 6:**
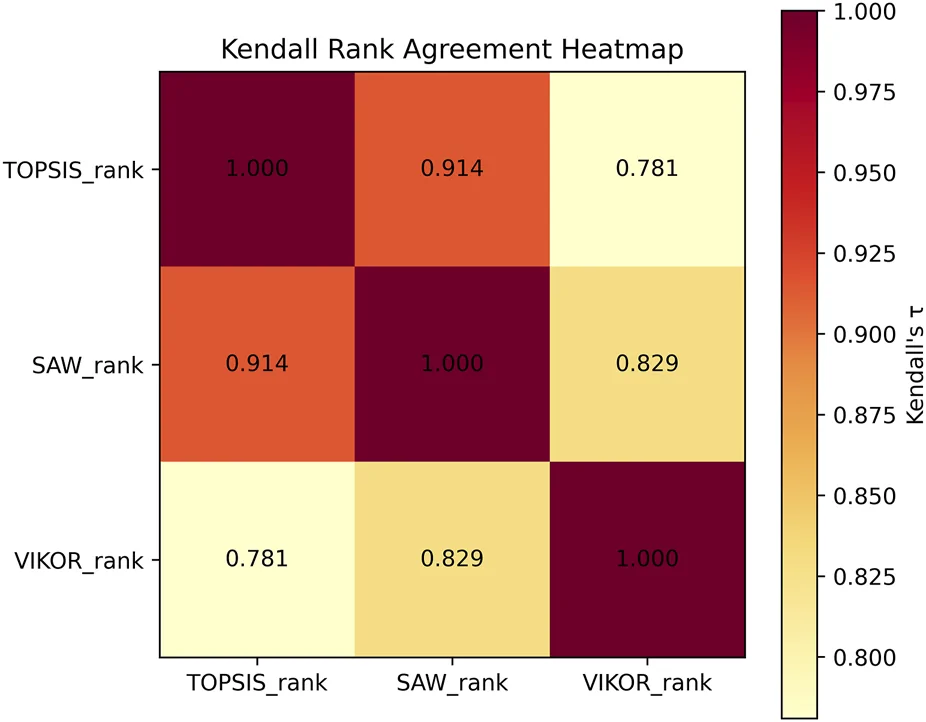
Heatmap corresponding to Kendall rank correlation.

Further insight into the similarity of the rankings is provided by the rank correlation analyses. The Spearman correlation coefficients (Table 9) range from 0.914 to 0.983, while the corresponding Kendall’s tau coefficients (Figure 6) vary between 0.781 and 0.914, with all correlations being statistically significant (p<0.001) . These values indicate a strong positive association among the rankings produced by TOPSIS, SAW, and VIKOR.

**TABLE 9:** Spearman rank correlation.

​	TOPSIS rank	SAW rank	VIKOR rank
TOPSIS rank	1	0.983117	0.914286
SAW rank	0.983117	1	0.946753
VIKOR rank	0.914286	0.946753	1

The average rank difference (ARD) values reported in Table 10 provide additional evidence of the similarity between the ranking methods. The ARD values range from 0.54 to 1.17 rank positions, corresponding to only 2.57%–5.59% of the total ranking range. Furthermore, the mean pairwise rank differences do not exceed two positions, indicating that the relative ordering of the compounds is largely preserved across the different MCDM techniques.

**TABLE 10:** Summary of ARD analysis.

Average rank difference	
TOPSIS rank	0.8254
SAW rank	0.5397
VIKOR rank	1.1746
TOPSIS rank vs. SAW rank: 0.762	
TOPSIS rank vs. VIKOR rank: 1.905	
SAW rank vs. VIKOR rank: 1.619	

Among the three pairwise comparisons, TOPSIS and SAW exhibit the closest agreement, with a Spearman correlation coefficient of 0.983, Kendall’s tau coefficient of 0.914, and an average rank difference of only 0.76 positions. In contrast, VIKOR displays slightly different ranking behavior, which may be attributed to its compromise-solution philosophy that simultaneously considers group utility and individual regret. Nevertheless, the observed differences are relatively small, and all three methods lead to broadly consistent conclusions regarding the prioritization of the investigated UV-filter compounds.

## 5 Discussion

The present study demonstrates how concepts from chemical graph theory can be employed to investigate the physicochemical behavior of UV-filter compounds from a purely structural perspective. By representing molecules as graphs and extracting degree-based descriptors and their corresponding graph energies, it becomes possible to establish quantitative links between molecular topology and experimentally measured properties without requiring extensive laboratory experimentation. The computational framework adopted in this work further illustrates the practicality of integrating graph-theoretical descriptors, statistical modeling, validation procedures, and decision-making techniques within a single reproducible workflow.

An important observation arising from the study is the difference in predictive behavior among the investigated physicochemical properties. Molecular weight, complexity, refractivity, and polarizability were consistently described more effectively than XlogP, topological polar surface area, and aqueous solubility. This behavior can be interpreted from the nature of the descriptors themselves. Degree-based topological indices are primarily determined by molecular size, branching pattern, and atomic connectivity. Properties such as molecular weight and complexity are therefore closely related to the structural information encoded within the molecular graph. Similarly, refractivity and polarizability are influenced by the overall size and electronic distribution of a molecule, which often correlate with the extent of molecular connectivity. In contrast, properties such as lipophilicity and aqueous solubility depend not only on molecular topology but also on intermolecular interactions, hydrogen-bonding effects, and other physicochemical factors that are not fully captured by simple degree-based descriptors. Consequently, weaker structure-property relationships for XlogP and LogS are not unexpected.

It should be noted that the strong correlations observed for molecular weight are also not unexpected, as many degree-based topological descriptors inherently increase with molecular size. Therefore, these correlations should be interpreted as reflecting the size dependence of the descriptors rather than their superior predictive capability, making their performance for other physicochemical properties more informative in assessing descriptor effectiveness.

Another noteworthy outcome is the close similarity observed between the predictive performances of the conventional topological indices and their corresponding graph-energy descriptors. Since graph energies are derived from spectra of matrices whose entries are themselves based on degree-related information, both classes of descriptors ultimately encode similar structural characteristics. The relatively small differences observed between the two descriptor families suggest that the spectral transformation preserves much of the information contained in the original indices. This observation supports the use of graph-energy descriptors as complementary molecular descriptors in QSPR investigations while also indicating that, for the present dataset, the underlying degree information remains the dominant factor governing predictive performance.

The validation results provide additional insight into the stability of the developed models. Although individual train-test partitions occasionally produced lower predictive coefficients, the overall agreement among regression analysis, leave-one-out cross-validation, and Monte Carlo validation indicates that the identified trends are not artifacts of a particular statistical procedure. The consistency observed across multiple validation approaches suggests that the relationships identified for the well-performing properties are sufficiently robust to warrant further investigation on larger datasets.

From a practical perspective, the ranking analysis provides a complementary view of the investigated compounds. While QSPR modeling focuses on understanding and predicting individual physicochemical properties, MCDM methods facilitate the simultaneous evaluation of multiple criteria. The strong agreement observed among TOPSIS, SAW, and VIKOR suggests that the prioritization of compounds is relatively insensitive to the choice of ranking algorithm. This is particularly important because different MCDM methods rely on distinct mathematical principles; therefore, convergence toward similar rankings increases confidence in the resulting prioritization. The use of CRITIC weighting further strengthens this analysis by assigning importance to criteria based on both their variability and the information contained in their mutual relationships, thereby reducing the influence of subjective weighting choices.

Finally, the study highlights the broader potential of combining chemical graph theory with data-driven decision-making approaches. Although degree-based descriptors alone cannot capture every aspect of molecular behavior, they provide computationally inexpensive structural information that can be readily incorporated into predictive and screening frameworks. When coupled with statistical validation and multi-criteria evaluation techniques, such descriptors offer a useful foundation for the preliminary assessment of chemical compounds and may assist in identifying promising candidates for more detailed experimental investigation.

### 5.1 Implications

Future studies may extend the present work by considering larger and more structurally diverse classes of UV-filters, additional molecular descriptors, and advanced machine-learning approaches capable of capturing nonlinear structure-property relationships. The incorporation of toxicological, environmental, photostability, and formulation-related parameters into the decision-making framework may also provide a more comprehensive assessment of UV-filter performance. Such investigations could contribute to the identification and development of safer and more effective photoprotective agents for future applications.

### 5.2 Limitations and future perspectives

The analysis was restricted to a relatively small set of commercially relevant UV-filter compounds and a limited number of physicochemical properties. Owing to the limited dataset size, an independent external validation set could not be established, and model validation was therefore confined to internal validation techniques. Furthermore, only single-descriptor linear QSPR models were considered, and the ranking procedure relied exclusively on the selected physicochemical criteria. Consequently, the conclusions should be interpreted within the scope of the investigated dataset and descriptor set. Future studies involving larger and more diverse datasets will facilitate rigorous external validation, while the exploration of nonlinear, multivariate, and machine-learning approaches may further improve the predictive capability and generalizability of the proposed models.

## 6 Conclusion

In this work, the applicability of selected degree-based topological indices and their corresponding graph-energy descriptors was investigated for modeling the physicochemical properties of UV-filter compounds. Correlation analysis, regression modeling, leave-one-out cross-validation, and Monte Carlo validation were employed to assess the predictive capability of the proposed descriptors. The results indicate that both classes of descriptors are strongly associated with molecular weight, complexity, refractivity, and polarizability, whereas weaker relationships were observed for XlogP, topological polar surface area, and aqueous solubility. In most cases, the conventional degree-based indices exhibited slightly better predictive performance than their corresponding graph energies, although the differences were generally small.

The validation analyses revealed that the developed models maintain satisfactory predictive performance across different evaluation strategies, particularly for molecular weight, complexity, refractivity, and polarizability. These findings suggest that the investigated descriptors capture structural information relevant to these properties and may serve as useful tools for QSPR studies involving UV-filter compounds. The subsequent MCDM analysis provided a systematic ranking of the investigated compounds based on their physicochemical characteristics. Despite methodological differences among TOPSIS, SAW, and VIKOR, the rankings showed strong agreement, leading to a consistent prioritization of the most promising candidates. In particular, Diethylhexyl Butamido Triazone, Ethylhexyl Triazone, and Bisoctrizole emerged as the highest-ranked compounds according to the adopted criteria.

## Data Availability

The original contributions presented in the study are included in the article/Supplementary Material, further inquiries can be directed to the corresponding author.
