# Supplementary material for: Degree-based topological indices and their graph energies in the QSPR analysis and ranking of UV-filter compounds
Source: Front Chem. 2026 Aug 14;14:1908317. doi: 10.3389/fchem.2026.1908317 (PMC13522127; doi:10.3389/fchem.2026.1908317)
Supplement: Supplementary file 1 [file DataSheet1.pdf]

Supplementary Material
Python Codes Used for the article:
Degree-Based Topological Indices and Their Graph Energies in the QSPR Analysis and Ranking of UV-Filter Compounds

July 18, 2026

Contents

1 Introduction **2**

2 Calculation of Degree Based Topological Indices **2**

3 Calculation of Graph Energy Descriptors **5**

4 Linear Regression & LOO-CV **10**

5 Monte Carlo Validation **12**

6 Scatter & Residual Plots **14**

7 MCDM Ranking **16**

1 Introduction

This document provides the Python scripts used to compute descriptors, linear regression modeling, and validation processes. The file uv.csv has the SMILES representation of the UV filters, which is provided in Table 1, and the file mcdm.csv contains all the physicochemical parameters of UV filters which is provided as 2.

name	SMILES
Avobenzone	CC(C)(C)C1=CC=C(C=C1)C(=O)CC(=O)C2=CC=C(C=C2)OC
Octocrylene	CCCCC(CC)COC(=O)C(=C(C1=CC=CC=C1)C2=CC=CC=C2)C#N
Octinoxate	CCCCC(CC)COC(=O)/C=C/C1=CC=C(C=C1)OC
Octisalate	CCCCC(CC)COC(=O)C1=CC=CC=C1O
Oxybenzone	COC1=CC(=C(C=C1)C(=O)C2=CC=CC=C2)O
Dioxybenzone	COC1=CC(=C(C=C1)C(=O)C2=CC=CC=C2O)O
Sulisobenzone	COC1=C(C=C(C(=C1)O)C(=O)C2=CC=CC=C2)S(=O)(=O)O
Ensulizole	C1=CC=C(C=C1)C2=NC3=C(N2)C=C(C=C3)S(=O)(=O)O
Homosalate	CC1CC(CC(C1)(C)C)OC(=O)C2=CC=CC=C2O
Padimate O	CCCCC(CC)COC(=O)C1=CC=C(C=C1)N(C)C
Meradimate	CC1CCC(C(C1)OC(=O)C2=CC=CC=C2N)C(C)C
Cinoxate	CCOCCOC(=O)/C=C/C1=CC=C(C=C1)OC
Ecamsule	CC1(C2CCC1(C(=O)C2=CC3=CC=C(C=C3)C=C4C5CCC(C4=O)(C5(C)C)CS(=O)(=O)O)CS(=O)(=O)O)C
Drometrizole Trisiloxane	CC1=CC(=C(C(=C1)N2N=C3C=CC=CC3=N2)O)CC(C)C[Si](C)(O[Si](C)(C)C)O[Si](C)(C)C
Diethylhexyl Butamido Triazone	CCCCC(CC)COC(=O)C1=CC=C(C=C1)NC2=NC(=NC(=N2)NC3=CC=C(C=C3)C(=O)NC(C)(C)C)NC4=CC=C(C=C4)C(=O)OCC(CC)CCCC
Ethylhexyl Triazone	CCCCC(CC)COC(=O)C1=CC=C(C=C1)NC2=NC(=NC(=N2)NC3=CC=C(C=C3)C(=O)OCC(CC)CCCC)NC4=CC=C(C=C4)C(=O)OCC(CC)CCCC
Diethylamino Hydroxybenzoyl Hexyl Benzoate	CCCCCCOC(=O)C1=CC=CC=C1C(=O)C2=C(C=C(C=C2)N(CC)CC)O
Bisoctrizole	CC(C)(C)CC(C)(C)C1=CC(=C(C(=C1)N2N=C3C=CC=CC3=N2)O)CC4=C(C(=CC(=C4)C(C)(C)CC(C)(C)C)N5N=C6C=CC=CC6=N5)O
Bemotrizinol	CCCCC(CC)COC1=CC(=C(C=C1)C2=NC(=NC(=N2)C3=CC=C(C=C3)OC)C4=C(C=C(C=C4)OCC(CC)CCCC)O)O
Amiloxate	CC(C)CCOC(=O)/C=C/C1=CC=C(C=C1)OC
4-Methylbenzylidene Camphor	CC1=CC=C(C=C1)/C=C/2 \\C3CCC(C2=O)(C3(C)C)C

Table 1: Content of uv.csv

Molecular Weight	XLogP	Complexity	logS	Polar Surface Area	Refractivity	Polarizability
310.4	4.8	405	-3.2	66.76	67.06	24.81
361.5	7.1	510	-5.4	50.09	118.58	41.43
290.4	5.3	304	-5.8	35.53	86.44	34.84
250.33	5.7	240	-4	46.53	72.21	28.8
228.24	3.6	258	-3.2	46.53	65.08	23.96
244.24	3.3	292	-3.2	66.76	67.06	24.81
308.31	2.2	462	-3.2	100.9	75.7	29.14
274.3	2	414	-2.9	83.05	80.69	27.73
262.34	5	324	-3.8	46.53	74.65	29.86
277.4	5	270	-3.9	29.54	84.66	33.73
275.4	5.1	329	-4.7	52.32	81.97	31.56
250.29	2.4	255	-3.7	44.76	70.08	27.39
562.7	3.1	1230	-5	142.88	143.56	59.64
501.8	12.4	613	-5.8	69.4	137.97	54.24
766	11.8	1080	-5.9	156.46	223.95	87.63
823.1	14.5	1030	-6.7	153.66	240.34	97.67
397.5	6.5	505	-4.8	66.84	117.64	45.58
658.9	12.8	989	-6.2	101.88	220.17	75.8
627.8	10.4	761	-6.3	106.82	216.4	75.71
248.32	3.9	263	-4.5	35.53	72.71	28.33
254.4	4.5	423	-4.7	17.07	79.68	30.58

Table 2: Content of mcdm.csv

2 Calculation of Degree Based Topological Indices

```
pip install mordred rdkit

#Convert SMILES to Molecular Graph

```

```python
from rdkit import Chem
import networkx as nx
import numpy as np

def mol_to_graph(smiles):
    mol = Chem.MolFromSmiles(smiles)

    G = nx.Graph()

    for atom in mol.GetAtoms():
        G.add_node(atom.GetIdx())

    for bond in mol.GetBonds():
        u = bond.GetBeginAtomIdx()
        v = bond.GetEndAtomIdx()
        G.add_edge(u, v)

    return G

###first zagreb

def zagreb1(G):
    return sum(d*d for _, d in G.degree())

###second zagreb

def zagreb2(G):
    s = 0
    for u, v in G.edges():
        s += G.degree(u)*G.degree(v)
    return s

###randic

import math

def randic(G):
    s = 0
    for u, v in G.edges():
        s += 1/math.sqrt(G.degree(u)*G.degree(v))
    return s

###harmonic

def harmonic(G):
    s = 0
    for u, v in G.edges():
        s += 2/(G.degree(u)+G.degree(v))
    return s

###abc
```

```python

def abc(G):

    s = 0

    for u,v in G.edges():

        du = G.degree(u)
        dv = G.degree(v)

        s += ((du+dv-2)/(du*dv))**0.5

    return s

###ga

def ga(G):

    s = 0

    for u,v in G.edges():

        du = G.degree(u)
        dv = G.degree(v)

        s += (2*math.sqrt(du*dv))/(du+dv)

    return s

###sombor

def sombor(G):

    s = 0

    for u,v in G.edges():

        du = G.degree(u)
        dv = G.degree(v)

        s += math.sqrt(du**2 + dv**2)

    return s

###forgotten

def f_index(G):
    return sum(d**3 for _, d in G.degree())

#
    ######################################################################
```

```python

import pandas as pd

df = pd.read_csv("uv.csv")

results = []

for _, row in df.iterrows():

    G = mol_to_graph(row["SMILES"])

    results.append({
        "Name": row["name"],
        "M1": zagreb1(G),
        "M2": zagreb2(G),
        "Randic": randic(G),
        "Harmonic": harmonic(G),
        "ABC": abc(G),
        "GA": ga(G),
        "Sombor": sombor(G),
        "Forgotten": f_index(G)
    })

out = pd.DataFrame(results)

#out.to_csv("topological_indices.csv", index=False)
out.to_excel(
    "topological_indices.xlsx",
    index=False,
    engine="openpyxl"
)

print(out.head())
```

Listing 1: Calculation of DTIs

3 Calculation of Graph Energy Descriptors

```python
#Convert SMILES to TI-matrix energy

from rdkit import Chem
import networkx as nx
import numpy as np
import pandas as pd

def mol_to_graph(smiles):
    mol = Chem.MolFromSmiles(smiles)

    G = nx.Graph()
```

```python

    for atom in mol.GetAtoms():
        G.add_node(atom.GetIdx())

    for bond in mol.GetBonds():
        u = bond.GetBeginAtomIdx()
        v = bond.GetEndAtomIdx()
        G.add_edge(u, v)

    return G

def matrix_energy(M):
    eigvals = np.linalg.eigvals(M)
    return np.sum(np.abs(eigvals))

def zagreb1_energy(G):

    n = G.number_of_nodes()
    nodes = list(G.nodes())

    Z1 = np.zeros((n,n))

    for u,v in G.edges():

        i = nodes.index(u)
        j = nodes.index(v)

        val = G.degree(u) + G.degree(v)

        Z1[i,j] = val
        Z1[j,i] = val

    return matrix_energy(Z1)

def zagreb2_energy(G):

    n = G.number_of_nodes()
    nodes = list(G.nodes())

    Z2 = np.zeros((n,n))

    for u,v in G.edges():

        i = nodes.index(u)
        j = nodes.index(v)

        val = G.degree(u)*G.degree(v)

        Z2[i,j] = val
        Z2[j,i] = val

```

```
    return matrix_energy(Z2)

def randic_energy(G):

    n = G.number_of_nodes()
    nodes = list(G.nodes())

    R = np.zeros((n,n))

    for u,v in G.edges():

        i = nodes.index(u)
        j = nodes.index(v)

        val = 1/np.sqrt(G.degree(u)*G.degree(v))

        R[i,j] = val
        R[j,i] = val

    return matrix_energy(R)

def harmonic_energy(G):

    n = G.number_of_nodes()
    nodes = list(G.nodes())

    H = np.zeros((n,n))

    for u,v in G.edges():

        i = nodes.index(u)
        j = nodes.index(v)

        val = 2/(G.degree(u)+G.degree(v))

        H[i,j] = val
        H[j,i] = val

    return matrix_energy(H)

def abc_energy(G):

    n = G.number_of_nodes()
    nodes = list(G.nodes())

    A = np.zeros((n,n))

    for u,v in G.edges():

        i = nodes.index(u)
        j = nodes.index(v)
```

```

        du = G.degree(u)
        dv = G.degree(v)

        val = np.sqrt((du+dv-2)/(du*dv))

        A[i,j] = val
        A[j,i] = val

    return matrix_energy(A)

def ga_energy(G):

    n = G.number_of_nodes()
    nodes = list(G.nodes())

    GA = np.zeros((n,n))

    for u,v in G.edges():

        i = nodes.index(u)
        j = nodes.index(v)

        du = G.degree(u)
        dv = G.degree(v)

        val = (2*np.sqrt(du*dv))/(du+dv)

        GA[i,j] = val
        GA[j,i] = val

    return matrix_energy(GA)

def sombor_energy(G):

    n = G.number_of_nodes()
    nodes = list(G.nodes())

    S = np.zeros((n,n))

    for u,v in G.edges():

        i = nodes.index(u)
        j = nodes.index(v)

        val = np.sqrt(G.degree(u)**2 + G.degree(v)**2)

        S[i,j] = val
        S[j,i] = val

    return matrix_energy(S)
```

```python

def f_energy(G):

    nodes = list(G.nodes())
    idx = {node:i for i,node in enumerate(nodes)}

    n = len(nodes)

    F = np.zeros((n,n))

    for u,v in G.edges():

        i = idx[u]
        j = idx[v]

        du = G.degree(u)
        dv = G.degree(v)

        val = du**2 + dv**2

        F[i,j] = val
        F[j,i] = val

    return matrix_energy(F)

df = pd.read_csv("uv.csv")

results = []

for _, row in df.iterrows():

    G = mol_to_graph(row["SMILES"])

    results.append({

        "Name": row["name"],

        "M1_Energy": zagreb1_energy(G),
        "M2_Energy": zagreb2_energy(G),
        "Randic_Energy": randic_energy(G),
        "Harmonic_Energy": harmonic_energy(G),
        "ABC_Energy": abc_energy(G),
        "GA_Energy": ga_energy(G),
        "Sombor_Energy": sombor_energy(G),
        "F_Energy": f_energy(G)

    })

out = pd.DataFrame(results)

out.to_excel(
```

```
    "matrix energies.xlsx",
    index=False,
    engine="openpyxl"
)

print(out.head())
```

Listing 2: Calculation of GEDs

4 Linear Regression & LOO-CV

Python code for the calculation of statistical parameters for the linear regression and Leave-one-out cross validation for molecular complexity. Same code follows for all other physicochemical properties with values of the parameters replaced with corresponding values.

```
import numpy as np
import statsmodels.api as sm
from sklearn.linear_model import LinearRegression
from sklearn.model_selection import LeaveOneOut
from sklearn.metrics import r2_score, mean_squared_error,
   mean_absolute_error
import pandas as pd

x = np.array([
    312,302,216,192,210,230,296,282,268,220,258,
    180,708,514,700,702,342,798,566,192,324
], dtype=float)

y = np.array([
    405,510,304,240,258,292,462,414,324,270,329,
    255,1230,613,1080,1030,505,989,761,263,423
], dtype=float)
X = x.reshape(-1,1)
# =====================================================
# FIT FULL MODEL
# =====================================================

X_sm = sm.add_constant(X)

model = sm.OLS(y, X_sm).fit()

a = model.params[0]
b = model.params[1]

print("\n==============================")
print("LINEAR MODEL")
print("==============================")

print(f"Equation: y = {a:.6f} + {b:.6f}x")

```

```python
print(f"R^2             = {model.rsquared:.6f}")
print(f"Adjusted R^2    = {model.rsquared_adj:.6f}")
print(f"F-statistic    = {model.fvalue:.6f}")
print(f"Model p-value = {model.f_pvalue:.6e}")

print("\nCoefficient Statistics")
print("----------------------")

for name, coef, pval in zip(
    ["Intercept","Slope"],
    model.params,
    model.pvalues
):
    print(f"{name}:")
    print(f"   Coefficient = {coef:.6f}")
    print(f"   p-value     = {pval:.6e}")

# ====================================================
# RMSE & MAE
# ====================================================

y_fit = model.predict(X_sm)

rmse = np.sqrt(mean_squared_error(y, y_fit))
mae = mean_absolute_error(y, y_fit)

print("\nError Statistics")
print("----------------------")
print(f"RMSE = {rmse:.6f}")
print(f"MAE  = {mae:.6f}")

# ====================================================
# LOO CROSS VALIDATION
# ====================================================

loo = LeaveOneOut()

y_cv = np.zeros(len(y))

for train_idx, test_idx in loo.split(X):

    X_train = X[train_idx]
    y_train = y[train_idx]

    X_test = X[test_idx]

    lr = LinearRegression()
    lr.fit(X_train, y_train)

    y_cv[test_idx] = lr.predict(X_test)

```

```
PRESS = np.sum((y - y_cv)**2)

TSS = np.sum((y - np.mean(y))**2)

Q2_LOO = 1 - PRESS/TSS

RMSECV = np.sqrt(
    mean_squared_error(y, y_cv)
)

print("\nLOO-CV Validation")
print("----------------------")
print(f"Q^2_LOO = {Q2_LOO:.6f}")
print(f"RMSECV = {RMSECV:.6f}")


results = {
    "R2": model.rsquared,
    "Adj_R2": model.rsquared_adj,
    "F_stat": model.fvalue,
    "F_pvalue": model.f_pvalue,
    "RMSE": rmse,
    "MAE": mae,
    "Intercept": model.params[0],
    "Slope": model.params[1],
    "Q2-LOO": Q2_LOO,
    "RMSECV": RMSECV
}

pd.DataFrame([results]).to_excel(
    "OLS_results_CX.xlsx",
    index=False
)
```

Listing 3: Regression & LOO-CV for complexity

5 Monte Carlo Validation

Python code for the Monte Carlo Validation of molecular complexity. Same code follows for all other physicochemical properties with values of the parameters replaced with corresponding values.

```
import numpy as np
from sklearn.linear_model import LinearRegression
from sklearn.model_selection import train_test_split
from sklearn.metrics import r2_score, mean_squared_error


# INPUT DATA

# ---complexity \& forgotten---
```

```
x = np.array([
    312,302,216,192,210,230,296,282,268,220,258,
    180,708,514,700,702,342,798,566,192,324
], dtype=float)

y = np.array([
    405,510,304,240,258,292,462,414,324,270,329,
    255,1230,613,1080,1030,505,989,761,263,423
], dtype=float)

X = x.reshape(-1,1)

#
# MONTE CARLO CROSS VALIDATION
#

n_iterations = 500
test_size = 0.20

r2_list = []
rmsep_list = []

for i in range(n_iterations):

    X_train, X_test, y_train, y_test = train_test_split(
        X,
        y,
        test_size=test_size,
        random_state=i
    )

    model = LinearRegression()
    model.fit(X_train, y_train)

    y_pred = model.predict(X_test)

    r2 = r2_score(y_test, y_pred)

    rmsep = np.sqrt(
        mean_squared_error(y_test, y_pred)
    )

    r2_list.append(r2)
    rmsep_list.append(rmsep)

#
# RESULTS
#

r2_mean = np.mean(r2_list)
r2_sd = np.std(r2_list)
```

```python

rmsep_mean = np.mean(rmsep_list)
rmsep_sd = np.std(rmsep_list)


print("MONTE CARLO VALIDATION")


print(f"Iterations       : {n_iterations}")
print(f"Train/Test Split : 80/20")

print("\nR^2pred")
print(f"Mean = {r2_mean:.6f}")
print(f"SD   = {r2_sd:.6f}")
print(f"Min  = {min(r2_list):.6f}")
print(f"Max  = {max(r2_list):.6f}")

print("\nRMSEP")
print(f"Mean = {rmsep_mean:.6f}")
print(f"SD   = {rmsep_sd:.6f}")
print(f"Min  = {min(rmsep_list):.6f}")
print(f"Max  = {max(rmsep_list):.6f}")

good = np.sum(np.array(r2_list) > 0.5)
excellent = np.sum(np.array(r2_list) > 0.8)
negative = np.sum(np.array(r2_list) < 0)

print("R^2pred > 0.5 :", good)
print("R^2pred > 0.8 :", excellent)
print("Negative R^2pred :", negative)

print("Percentage > 0.5 :", 100*good/500)
print("Percentage > 0.8 :", 100*excellent/500)
print("Percentage negative :", 100*negative/500)
```

Listing 4: Monte Carlo validation for complexity

6 Scatter & Residual Plots

Python code for the Scatter & residual plots for molecular weight. Same code follows for all other physicochemical properties with values of the parameters replaced with corresponding values.

```python
import pandas as pd
import numpy as np
import matplotlib.pyplot as plt
import seaborn as sns

from sklearn.linear_model import LinearRegression
from sklearn.metrics import mean_squared_error,
    mean_absolute_error, r2_score
```

```python
import statsmodels.api as sm

### MW & ABC
data = {
    'TopologicalIndex': [17.52360843,19.74661921,14.9586322,12.86582143,12.82538132,
    13.60143778,16.02851464,15.18350849,14.70726204,14.38942479,15.23443095,
    12.83731186,30.90878326,26.02757664,42.40290501,44.75457627,
    21.29873215,39.54499198,34.66481131,13.05609146,15.24594418
],
    'Property': [310.4,361.5,290.4,250.33,228.24,244.24,308.31,274.3,262.34,
    277.4,275.4,250.29,562.7,501.8,766,823.1,397.5,658.9,627.8,248.32,254.4
]}
df = pd.DataFrame(data)

# Independent and dependent variables
X = df[['TopologicalIndex']]
y = df['Property']

#  Linear Regression using sklearn
model = LinearRegression()
model.fit(X, y)
y_pred = model.predict(X)

#  Residual Plot
import seaborn as sns
residuals = y - y_pred
sns.set(style='whitegrid')
sns.residplot(x=y_pred, y=residuals, color='blue')
#plt.figure(figsize=(8,5))
#plt.scatter(y_pred, residuals, color='blue', edgecolor='k', alpha=0.7)
#plt.axhline(y=0, color='red', linestyle='--', linewidth=1.5, label='Residual = 0')
plt.xlabel('Predicted')
plt.ylabel('Residuals')
plt.title('Molecular Weight - ABC Index')
plt.savefig('residual_MW1.png', dpi=600, bbox_inches='tight')
plt.show()

# Linear regression fit
plt.figure(figsize=(8, 5))
plt.scatter(X, y, color='blue', label='Actual Data')
plt.plot(X, y_pred, color='red', label='Fitted Line')
plt.xlabel('ABC Index')
plt.ylabel('Molecular Weight')
```

```
plt.title('')
plt.legend()
plt.grid(True)
plt.savefig('scatter_MW1.png', dpi=600, bbox_inches='tight')
plt.show()
```

Listing 5: Plots for molecular weight

7 MCDM Ranking

```
df = pd.read_csv("mcdm.csv")

id_cols = df.select_dtypes(exclude=[np.number])
X = df.select_dtypes(include=[np.number])

def normalize(df):
    return (df - df.min()) / (df.max() - df.min() + 1e-12)

X_norm = normalize(X)

def entropy_weights(df):
    P = df / (df.sum() + 1e-12)

    n, m = df.shape
    k = 1 / np.log(n)

    e = np.zeros(m)

    for j in range(m):
        p = P.iloc[:, j].replace(0, 1e-12)
        e[j] = -k * np.sum(p * np.log(p))

    d = 1 - e
    return d / d.sum()

def critic_weights(df):
    std = df.std()

    corr = df.corr().abs()

    C = []

    for j in df.columns:
        corr_sum = corr[j].sum() - 1  # exclude self-correlation
        Cj = std[j] * (1 - (corr_sum / (len(df.columns) - 1)))
        C.append(Cj)

    C = np.array(C)
    return C / C.sum()

```

```
def hybrid_weights(df, alpha=0.5):
    w_entropy = entropy_weights(df)
    w_critic = critic_weights(df)

    hybrid = alpha * w_entropy + (1 - alpha) * w_critic

    return hybrid / hybrid.sum()

weights = hybrid_weights(X_norm)
weights = pd.Series(weights, index=X.columns)

print(weights)

def topsis(df, weights, benefit_cols):
    norm = df / np.sqrt((df**2).sum())
    weighted = norm * weights

    ideal_best = {}
    ideal_worst = {}

    for col in df.columns:
        if col in benefit_cols:
            ideal_best[col] = weighted[col].max()
            ideal_worst[col] = weighted[col].min()
        else:
            ideal_best[col] = weighted[col].min()
            ideal_worst[col] = weighted[col].max()

    ideal_best = pd.Series(ideal_best)
    ideal_worst = pd.Series(ideal_worst)

    d_plus = np.sqrt(((weighted - ideal_best) ** 2).sum(axis=1))
    d_minus = np.sqrt(((weighted - ideal_worst) ** 2).sum(axis=1))

    return d_minus / (d_plus + d_minus)

def saw(df, weights, benefit_cols):
    norm = df.copy()

    for col in df.columns:
        if col in benefit_cols:
            norm[col] = df[col] / df[col].max()
        else:
            norm[col] = df[col].min() / df[col]

    return (norm * weights).sum(axis=1)

def vikor(df, weights, benefit_cols, v=0.5):

    f_star = {}
    f_minus = {}
```

```

    for col in df.columns:
        if col in benefit_cols:
            f_star[col] = df[col].max()
            f_minus[col] = df[col].min()
        else:
            f_star[col] = df[col].min()
            f_minus[col] = df[col].max()

    S = []
    R = []

    for i in range(len(df)):
        Si = 0
        Ri = 0

        for col in df.columns:
            fj = df.iloc[i][col]

            if col in benefit_cols:
                d = (f_star[col] - fj) / (f_star[col] - f_minus[col] + 1e-12)
            else:
                d = (fj - f_star[col]) / (f_minus[col] - f_star[col] + 1e-12)

            wd = weights[col] * d
            Si += wd
            Ri = max(Ri, wd)

        S.append(Si)
        R.append(Ri)

    S = np.array(S)
    R = np.array(R)

    Q = v * (S - S.min()) / (S.max() - S.min() + 1e-12) + \
        (1 - v) * (R - R.min()) / (R.max() - R.min() + 1e-12)

    return Q

benefit_cols = X.columns.tolist()  # or define properly

topsis_score = topsis(X_norm, weights, benefit_cols)
saw_score = saw(X_norm, weights, benefit_cols)
vikor_score = vikor(X_norm, weights, benefit_cols)

results = id_cols.copy().reset_index(drop=True)

results["TOPSIS"] = topsis_score
results["SAW"] = saw_score
```

```
results["VIKOR"] = vikor_score

results["TOPSIS_rank"] = results["TOPSIS"].rank(ascending=False)
results["SAW_rank"] = results["SAW"].rank(ascending=False)
results["VIKOR_rank"] = results["VIKOR"].rank(ascending=True)

results.to_excel("MCDM_CRITIC_ENTROPY.xlsx", index=False)

print(results.head())

### Spearman correlation

results[["TOPSIS_rank","SAW_rank","VIKOR_rank"]].corr(method="
    spearman")

### Kendall correlation

def kendall_matrix_with_p(df, rank_columns):

    tau_mat = pd.DataFrame(
        np.eye(len(rank_columns)),
        index=rank_columns,
        columns=rank_columns
    )

    p_mat = pd.DataFrame(
        np.zeros((len(rank_columns), len(rank_columns))),
        index=rank_columns,
        columns=rank_columns
    )

    for i in range(len(rank_columns)):
        for j in range(i+1, len(rank_columns)):

            tau, p = kendalltau(
                df[rank_columns[i]],
                df[rank_columns[j]]
            )

            tau_mat.iloc[i,j] = tau
            tau_mat.iloc[j,i] = tau

            p_mat.iloc[i,j] = p
            p_mat.iloc[j,i] = p

    return tau_mat, p_mat

tau_matrix, p_matrix = kendall_matrix_with_p(results, rank_cols)

print("Kendall Tau:")
print(tau_matrix)
```

```python

print("\nP-values:")
print(p_matrix)

### ARD Analysis

rank_cols = ["TOPSIS_rank", "SAW_rank", "VIKOR_rank"]
results["Average_Rank"] = results[rank_cols].mean(axis=1)
ard = {}

for col in rank_cols:
    ard[col] = np.mean(
        np.abs(results[col] - results["Average_Rank"])
    )

ard_df = pd.DataFrame.from_dict(
    ard,
    orient="index",
    columns=["ARD"]
)

print(ard_df)

n = len(results)

ard_df["ARD_%"] = 100 * ard_df["ARD"] / n

print(ard_df)

from itertools import combinations

for a, b in combinations(rank_cols, 2):
    diff = np.mean(np.abs(results[a] - results[b]))
    print(f"{a} vs {b}: {diff:.3f}")
```

Listing 6: Ranking of properties
